# Myocardial CT perfusion imaging for pre- and postoperative evaluation of myocardial ischemia in a patient with myocardial bridging: A case report: Erratum

**DOI:** 10.1097/MD.0000000000009105

**Published:** 2017-12-01

In the article, “Myocardial CT perfusion imaging for pre- and postoperative evaluation of myocardial ischemia in a patient with myocardial bridging: A case report”,^[1]^ which appeared in Volume 96, Issue 42 of *Medicine*, Dr. Kyung Eun Shin's affiliation should appear as “Division of Cardiovascular and Thoracic Imaging, Department of Radiology, Soonchunhyang University Hospital, Bucheon, Republic of Korea.”
